# Infant regulatory function acts as a protective factor for later traits of autism spectrum disorder and attention deficit/hyperactivity disorder but not callous unemotional traits

**DOI:** 10.1186/s11689-019-9274-0

**Published:** 2019-07-18

**Authors:** Rachael Bedford, Teodora Gliga, Alexandra Hendry, Emily J. H. Jones, Greg Pasco, Tony Charman, Mark H. Johnson, Andrew Pickles, The BASIS Team, Simon Baron-Cohen, Patrick Bolton, Bosiljka Milosavljevic, Susie Chandler, Mayada Elsabbagh, Janice Fernandes, Holly Garwood, Kristelle Hudry, Elizabeth Shephard, Leslie Tucker, Agnes Volein

**Affiliations:** 1 Biostatistics and Health Informatics Department, Institute of Psychiatry, Psychology & Neuroscience, King’s College London, London, UK; 2 Centre for Brain and Cognitive Development, Birkbeck College, University of London, London, UK; 3 Experimental Psychology Department, Oxford University, Oxford, UK; 4 Psychology Department, Institute of Psychiatry, Psychology & Neuroscience, King’s College London, London, UK; 5 Psychology Department, Cambridge University, Cambridge, UK

**Keywords:** Autism spectrum disorder, Attention deficit/hyperactivity disorder, Callous unemotional traits, Executive function, Infants at risk, Regulatory function

## Abstract

**Background:**

Reduced executive functions (EF) are commonly associated with developmental conditions (e.g., autism spectrum disorder, ASD; attention deficit/hyperactivity disorder, ADHD), although EF seems to be typical in children with callous unemotional (CU) traits. Regulatory function (RF) is a proposed infant precursor that maps on onto factors driving later EF. Here, we first test whether RF is specifically and negatively associated with ASD and ADHD traits, but not CU traits. Second, we test whether RF can act as a protective factor, by moderating the association between infant markers and subsequent ASD and ADHD traits.

**Methods:**

Participants were 79 infants at high (*N* = 42) and low (*N* = 37) familial risk for ASD. Data come from the 14-month infant visit (Autism Observational Scale for Infants; AOSI; activity level and RF from the Infant Behavior Questionnaire; IBQ) and the 7-year visit (ASD traits: Social Responsiveness Scale, SRS; ADHD traits: Conners 3, CU traits: Inventory of Callous Unemotional Traits).

**Results:**

Infant RF was negatively associated with later traits of ASD (*B* = − 0.5, *p* = 0.01) and ADHD inattention (*B* = − 0.24, *p* = 0.02) but not hyperactivity (*B* = − 0.25, *p* = 0.10) or CU traits (*B* = 0.02, *p* = 0.86). RF moderated the association between infant AOSI score and ASD traits, with a significant effect in those with low RF (*B* = 0.10, *p* = 0.006), not high RF (*B* = 0.01, *p* = 0.78). Similarly, for ADHD, infant activity level was associated with later ADHD inattention in those with low (*B* = 0.17, *p* = 0.04) but not high RF (*B* = 0.07, *p* = 0.48). For ADHD hyperactivity symptoms, activity level was predictive at both high and low levels of RF.

**Conclusions:**

Strong RF may allow children to compensate for other atypicalities, thus attenuating the association between infant markers and later disorder traits. Whilst infant RF was associated with both ASD and ADHD inattention traits, there was no association with ADHD hyperactivity or CU traits. This suggests that any protective effect may not be universal and emphasises the need for a better understanding of the underlying moderating mechanisms.

**Electronic supplementary material:**

The online version of this article (10.1186/s11689-019-9274-0) contains supplementary material, which is available to authorized users.

## Background

Executive functions (EF) refer to a set of skills including planning, online monitoring, inhibition and working memory, which support the ability to set and achieve goals [1]. EF difficulties are associated with a broad range of acquired and developmental disorders [2], including autism spectrum disorder (ASD [3]) and attention deficit/hyperactivity disorder (ADHD [4]). Johnson [5] argues that the co-occurrence between poor EF and some neurodevelopmental disorders could arise because young children with poor EF skills are less able to adapt to, or compensate for, atypicalities in other brain systems early in life. In typical development, EF emerges across a protracted period and is commonly only measured experimentally from around preschool age. Thus, in order to test for potential moderating effects of EF earlier in life, it is necessary to identify precursors to EF during infancy. One such proposed precursor [6] is infants’ regulatory function (RF), a set of processes that modulate an individual’s response/reactivity to a change in their environment.

Children with autism spectrum disorder (ASD) show high levels of co-occurring conditions including ADHD [7] and callous unemotional (CU) traits (Leno et al. 2015). In order to tease apart shared and distinct pathways, it is helpful to compare conditions with and without shared genetic overlap. There is high co-occurrence and overlapping genetic risk between ASD and attention deficit/hyperactivity disorder (ADHD [7–9]). Indeed, this has been taken by some to suggest a common developmental pathway [10]. Whilst children with high callous unemotional traits show some superficial phenotypic overlap with ASD, particularly in social-affective skills such as emotion recognition, the disorders have separate genetic aetiology [11]. This suggests different underlying developmental pathways. The current paper aims to test two key hypotheses. First, are atypicalities in infant RF specific to traits of particular disorders (i.e., ASD and ADHD), rather than a common shared factor? Second, does infant RF moderate the association between known infant markers and later disorder traits?

Difficulties in EF have been extensively observed amongst children and adults with autism spectrum disorder (ASD) [3], particularly for cognitive flexibility and the ability to co-ordinate multiple EF demands simultaneously [12]. Whilst EF is no longer considered likely to be the only causal mechanism involved in ASD, it may act as a modifier of the phenotype, which interacts with atypicalities in core domains such as social cognition, exacerbating or lessening symptom expression [5, 13]. ADHD has been linked most consistently to difficulties with inhibitory control, and the co-occurrence of ADHD symptoms in ASD is associated with lower performance on inhibition tasks [12, 14]. As with ASD, there is debate in the literature about the extent to which EF difficulties represent a core component of the disorder rather than a co-occurring component [15].

Whilst associated EF deficits are indeed common across different developmental disorders, they may not be universally affected in atypical development. Children with high callous unemotional (CU) traits are characterised by a lack of guilt, remorse and empathy; traits thought to map onto the construct of psychopathy [16, 17]. Very limited research has specifically investigated EF in individuals with high CU traits. Amongst children with ASD, who overall show difficulties with conflict monitoring [18], those with higher co-occurring CU traits are associated with relatively *superior* conflict monitoring [19]. Other studies show no difference in executive function performance between autistic children and adolescents with high versus low co-occurring CU traits [20, 21]. In the current paper, we test whether an infant precursor to later EF is associated specifically with ASD and ADHD traits, but not CU traits, in a population that is at increased risk for manifesting traits of all three of these conditions.

In order to test the associations between an infant precursor to EF and later emerging traits of developmental disorders, the current study uses a longitudinal, prospective sample of infants at familial risk for ASD. An estimated 20% of infants at high familial risk for ASD (who have an older sibling with an ASD diagnosis) go on to a clinical diagnosis of ASD themselves (e.g., [22]) and a further 20% show other developmental atypicalities: sub-clinical symptoms of ASD (referred to as the broader autism phenotype (BAP), low IQ scores, and symptoms of co-occurring disorders such as ADHD [23, 24]. At-risk sibling designs support the investigation of both clinical and subclinical (e.g., BAP) phenotypes of ASD; this may yield important insights into risk and protective mechanisms that are not afforded by classic case-control designs [25]. Further, prospective infant-sibling designs may reveal early differences that are later masked or complicated by intellectual ability, compensatory or secondary mechanisms, and interactions with co-occurring symptoms [26].

Hendry et al. [6] argue that infant RF maps onto factors driving child and adolescent EF. RF is captured by the regulatory capacity/orienting (RCO) composite from the Infant Behavior Questionnaire—Revised (IBQ-R) [27] and/or the effortful control composite of the Early Childhood Behavior Questionnaire [28] with toddlers. RCO measures infants’ ability to sustain and shift attention depending on environmental needs, their enjoyment of novelty and ability to recover from distress and their enjoyment of social closedness. From late infancy and beyond, RF is largely self-directed, but in the first year of life, RF is at least partially contingent on caregiver actions (i.e., an adult providing the means of distraction or offering a soothing cuddle) [29]. An important index of RF is thus the infant’s response to parent soothing. In toddlers, the effortful control composite of the Early Childhood Behavior Questionnaire [28] also captures the ability to plan and execute actions. Scores on the RCO composite in infancy predict effortful control scores at 18–22 months [30, 31] and 2–3 years of age [32, 33]. In turn, by the end of the second year of life, effortful control shows measurement and conceptual overlap with measures of EF [6].

Several studies have shown links between lower infant and toddler effortful control, including inhibitory control and later ADHD symptoms [34–36]. Similarly, in relation to later ASD outcome, previous infant-sibling studies have demonstrated that low parent-reported RF and effortful control are predictive of risk group membership and later ASD diagnosis from as early as 14 months [37, 38]. In line with this developmental timing, the executive attention network, which regulates orienting of attention and information processing, is thought to gain functionality towards the end of the first year of life [6, 39]. In order to understand the potential *moderating* effects of infant RF, here, we aim to test whether infant RF can moderate the association between known infant markers, which have previously been associated with ASD and ADHD traits.

Several early markers for ASD in infancy have been identified [40], which are precursors later symptomatology. Here, we chose a global measure of early autism-like behaviours, the Autism Observation Scale for Infants (AOSI [41]). This observational assessment measures behaviours including imitation, motor skills and the anticipation of social interaction. AOSI total scores measured when infants at-risk for ASD are aged 1 year are associated with later ASD symptoms and diagnostic outcome [42] and have previously been shown to be predictive of ASD outcome in the current cohort [43]. For ADHD, fewer infant markers have been identified, but one which may be specific to ADHD (as compared to ASD) in the current cohort is activity level [36] measured by the Infant Behavior Questionnaire—Revised (IBQ-R) [27]. Testing the effect of the interaction between AOSI scores and infant RF on later ASD symptoms and of the interaction between activity level and RF on later ADHD traits will enable us to test whether high RF has broad protective effects in relation to later outcome. Early markers of CU traits are less well established, and although some putative markers have previously been suggested (e.g., reduced face preference in 5-week-olds [44], and increased fearfulness in 14-month-olds [45]), these measures were not collected in the current cohort, and their specificity to later CU traits is also unknown.

In the current study, we first test the hypothesis that infant RF will be negatively associated with traits of ASD and ADHD, but not CU traits, predicting a significant difference in the strength of association between ASD, ADHD and CU traits. Second, we test the hypothesis that RF moderates the association between known infant marker (activity level for ADHD, and AOSI early autism-like behaviours for ASD) with later traits of ASD and ADHD [5].

## Method

### Participants

As part of the British Autism Study of Infant Siblings (BASIS: www.basisnetwork.org), 104 infants (54 high-risk, 21 male; 50 low-risk, 21 male) took part in a battery of assessments at 7 and 14 months and 2, 3 and 7 years. At enrolment, each high-risk (HR) infant (*n* = 54) had an older sibling (in 4 cases, a half-sibling) with a community clinical ASD diagnosis, confirmed using information from the *Development and Well-Being Assessment* (*DAWBA* [46]) and the *Social Communication Questionnaire* (*SCQ* [47]) by expert clinicians on our team (TC, PB)^1^. Low-risk (LR) controls (*n* = 50) were full-term infants (with one exception) recruited from a volunteer database at the Birkbeck Centre for Brain and Cognitive Development. For older siblings of LR infants, the SCQ was used to confirm the absence of ASD, with no child scoring above instrument cut-off (≥ 15; *n* = 1 missing data).

Of 53 HR and 48 LR children retained at the 3-year assessment, 44 HR (83%) and 37 LR (77%) agreed to take part in the follow-up study at 6–8 years. Of these, two HR children did not complete a research visit (parents completed questionnaires only). The retained sample did not differ from the non-retained sample in 3-year levels of ASD on the *Autism Diagnostic Observation Schedule—Generic* (ADOS-G [48]), *Social Responsiveness Scale—Second Edition* (*SRS-2* [49]) or SCQ, developmental level on the *Mullen Scales of Early Learning* [50], adaptive behaviour assessed with the *Vineland Adaptive Behavior Scales—Second Edition* (*VABS-II* [51]), or family income (all *p* > .4). The HR and LR groups did not differ in age (HR mean (SD), 90.8 (6.3) months; LR mean (SD), 89.3 (4.8) months; *t* (76) = − 1.13, *p* = .26) or sex (HR, 36.4% male; LR, 40.5% male; χ^*2*^ (1) = .15, *p* = .70) at the follow-up. The exact sample size differs between analyses depending on the missingness of independent variables, with *N* = 69 for the ASD regression model (HR = 36; LR = 33) and *N* = 76 for the ADHD models (HR = 41; LR = 35). Ethical approval was obtained from the NHS National Research Ethics Service (NHS RES London REC 08/H0718/76; 14/LO/0170). Parents provided written informed consent. At the mid-childhood visit, children provided written informed assent wherever possible given the developmental level.

### ASD outcome at 7 years of age (see [52] for full sample description)

To ascertain ASD diagnostic outcome according to DSM-5, four experienced researchers (ES, BM, GP, TC) reviewed information on ASD symptomatology (Autism Diagnostic Observation Schedule—Second Edition, ADOS-2, [53] *Autism Diagnostic Interview*—*Revised* ADI-R, [54] SCQ, for HR participants only) and adaptive functioning (VABS-II) and IQ (*Wechsler Abbreviated Scale of Intelligence*—*Second Edition*, *WASI-II*, [55]) for every HR and LR child. Fifteen HR children (7 boys, 8 girls) met DSM-5 (APA, 2013) criteria for ASD at age 7, and the remaining 27 HR children (8 boys, 19 girls) did not. The 2 HR children who completed only questionnaires were not categorised. None of the 37 LR children met the DSM-5 criteria for ASD, and none had a community clinical ASD diagnosis at 7 years. Group characteristics at age 7 are presented in Additional file 1: Table S1.

### Fourteen-month infant measures

*The Autism Observation Scale for Infants* (AOSI [41, 43]) is a semi-structured observational assessment of ASD behavioural markers in infancy collected at 7 and 14 months. In the current study, a 19-item version of the AOSI was used (see [56]) with items coded 0, 1, 2 or 3, which gives a total score (sum of all codes), with higher scores indexing greater atypicality. The majority of assessments were double coded with excellent reliability (*n* = 85, intraclass correlation coefficient = 0.95).

### Infant Behavior Questionnaire—Revised (IBQ-R) [27]

The IBQ-R (191 items) is a measure of temperament in which parents rate the frequency with which their infant exhibits particular behaviours in everyday contexts on a seven-point scale (never to always). Parents rate their child’s behaviour over the past week (IBQ-R). Data for the present analysis comes from the 14-month visit. Whilst Rothbart and colleagues conventionally recommend the use of the ECBQ for infants aged 13–17 months, they note that the IBQ may be more appropriate for use with samples with potential developmental delays [57]. IBQ-R items are averaged to yield subscales indexing different temperament dimensions, which show high continuity across instruments [33]. In the current study, *regulatory function* (*RF*) was chosen as an infant precursor to later emerging executive functions. Our measure of RF is the orienting/regulation factor subscale of the IBQ—which is a composite score of the duration of orienting, low intensity pleasure, cuddliness and soothability scales [27].

*Activity level* from the IBQ-R was also used as an infant marker for later emerging ADHD symptoms [36]. The activity subscale assesses limb movement, squirming and locomotor activity. Note that the items comprising the activity subscale are entirely different from those items used to assess RF (i.e., duration of orienting, low intensity pleasure, cuddliness and soothability scales).

### Seven-year outcome measures

#### Social Responsiveness Scale-2 (SRS-2 [49])

A parent-report measure of ASD symptoms (rated over the 6 months prior to testing), the *SRS-2* was chosen as the measure of ASD traits in the current analysis to be comparable with the parent-reported ADHD and CU trait measures. Age-normed SRS-2 T-scores were used (mean 50; SD 10; minimum-maximum ≤ 30 to ≥ 90).

#### Conners 3 ([58])

The parent-rated *Conners 3* was used to measure symptoms of ADHD (also rated over the 6 months before testing). T-scores (mean 50; SD 10; minimum-maximum score ≤ 30 to ≥ 90) for the inattention and hyperactivity/impulsivity domains were used in analyses.

#### Inventory of Callous Unemotional Traits (ICU [59])

The ICU is a parent report questionnaire with 24 items assessing uncaring, callous and unemotional behaviours. Each item is rated on a 4-point Likert scale from 0 (not at all true) to 3 (definitely true). The total score is used in the analysis.

### Statistical analysis

Descriptive statistics are presented in Tables 1 and 2. Analyses were performed in Stata [60]. The outcome variables—SRS T-score, Conners ADHD inattention and hyperactivity T-scores and ICU scores—were not normally distributed and were transformed using a lnskew0 transformation in Stata, which normalised all scales (Shapiro-Wilk test *p* values > 0.11). For regression models with interaction terms, all variables were mean centred.

First, a multivariate regression model was run to test whether RF was associated with ASD and ADHD (hyperactivity and inattention) but not CU traits. The ‘test’ command in Stata was used to run pairwise coefficient comparisons. Next, to test for a moderating role of RF, we ran multiple regressions in Stata. In the ASD regression model, we tested whether ASD symptoms (7-year SRS score) were predicted by 14-month AOSI score, 14 months RF, and their interaction, controlling for group (high versus low familial risk). In the ADHD models, we tested whether hyperactivity and inattention were predicted by 14-month activity, RF, and their interaction, again controlling for risk group. Because we hypothesised that the infant marker will be associated with later disorder traits only for those with low RF, we also planned to run separate regressions to test the association in those with high and low RF separately, again controlling for risk group.

**Table 1:** Infant risk and protective markers at 14 months: AOSI score, activity level and regulatory function

	High-risk	Low-risk	Overall
Early ASD-like behaviour (AOSI)			
Mean (SD)	4.88 (4.52)	3.31 (3.57)	4.14 (4.15)
*N*	41	36	77
Activity level (IBQ-R)			
Mean (SD)	4.27 (0.96)	4.010 (0.74)	4.19 (0.86)
*N*	41	35	76
Regulatory function (IBQ-R)			
Mean (SD)	4.47 (0.65)	4.81 (0.47)	4.63 (0.60)
*N*	41	35	76

*AOSI* Autism Observation Scale for Infants, *IBQ-R* Infant Behavior Questionnaire—revised

Table 2 shows the mean trait scores for ASD, ADHD and CU traits split by risk group. Correlations with the centred, skew-transformed trait scores show ASD traits were significantly positively correlated with both ADHD inattention and hyperactivity (*r* = 0.42, *p* < 0.001; *r* = 0.46, *p* < 0.001) and CU traits (*r* = 0.42, *p* < 0.001).

**Table 2:** ASD, ADHD and CU traits at 7 years

	High-risk	Low-risk	Overall
ASD traits (SRS-2)			
Mean (SD)	59.27 (19.63)	45.49 (5.82)	52.57 (16.11)
*N*	37	35	72
ADHD inattention (Conners 3)			
Mean (SD)	57.07 (13.95)	51.22 (9.40)	54.33 (12.32)
*N*	42	37	79
ADHD hyperactivity (Conners 3)			
Mean (SD)	59.26 (16.59)	52.16 (11.58)	55.94 (14.81)
*N*	42	37	79
Callous unemotional traits (ICU)			
Mean (SD)	21.83 (11.27)	18.09 (5.96)	20.17 (9.4)
*N*	40	32	72

Additional analyses are included in Additional file 1: Tables S2a, b, S3 and S4a–c: (1) correlations between RF and subdomains of ASD (social communication and RRBs) and CU traits (callous, unemotional, uncaring); (2) regression models re-run with Social Communication Questionnaire (SCQ) score as the outcome measure, because the SRS has been shown to relate to domain-general difficulties as well as ASD severity [61]; and (3) regression models which do not covary for risk group. Results from these additional and confirmatory analyses remain substantively similar.

## Results

### Regulatory function is associated with ASD and ADHD but not CU traits

A multivariate regression model showed a significant association between 14-month RF and later 7-year traits of ASD (*b* = − 0.497, S.E. = 0.197, *p* = 0.014), ADHD inattention (*b* = − 0.239, S.E. = 0.101, *p* = 0.021), though not hyperactivity (*b* = − 0.248, S.E. = 0.151, *p* = 0.104). No significant association was found between RF and later CU traits (*b* = 0.016, S.E. = 0.089, *p* = 0.861). Testing the difference between slopes showed that the association between RF and CU traits was significantly different to that for RF with ASD (*p* = 0.005) and ADHD inattention (*p* = 0.027), but not different to ADHD hyperactivity (*p* = 0.096).

### Does RF moderate the association between infant autism-like behaviours and later ASD symptoms at 7 years?

**Fig. 1:**
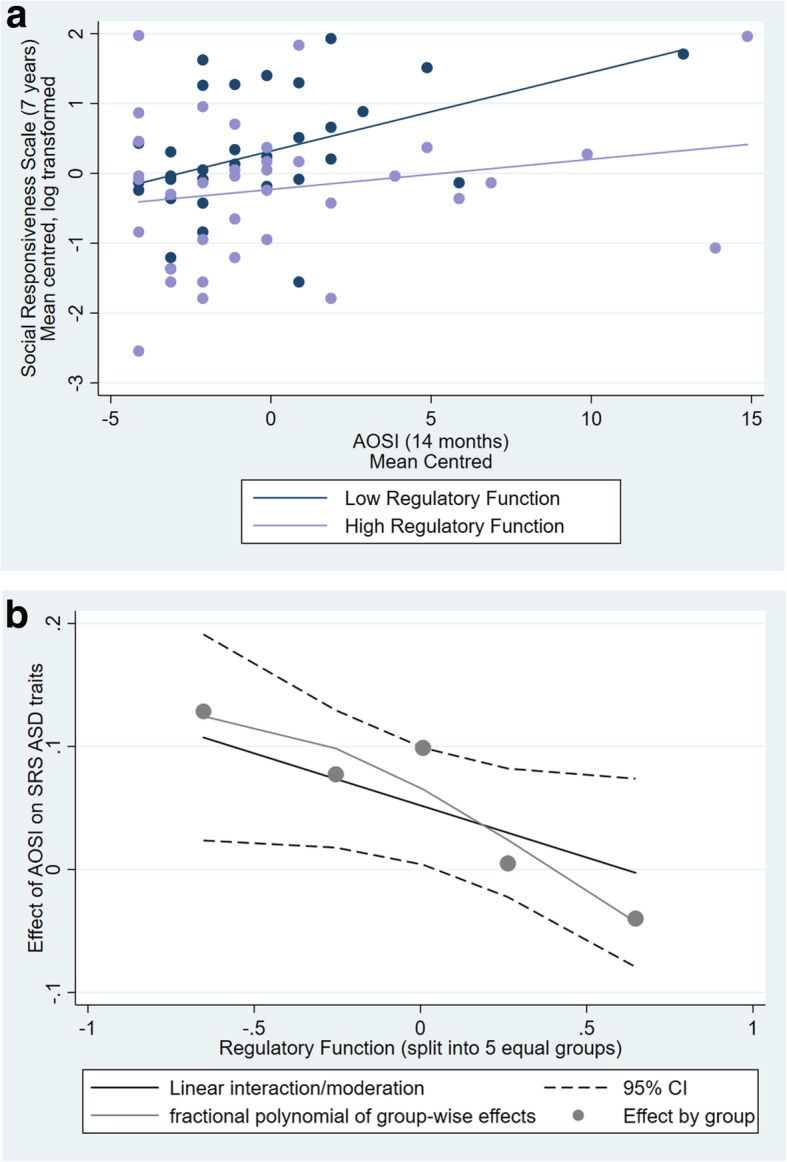
**a** The effect of infant AOSI score predicting later ASD traits on the SRS for those with high versus low RF. **b** The effect of RF (split into 5 equal groups) on the association between AOSI and SRS scores. At higher levels of RF, there is no association between AOSI and SRS, but at low RF levels, AOSI and SRS scores are positively associated

A multiple regression showed significant associations between 7-year SRS score and risk group (*b* = 0.799, S.E = 0.212, *p* < 0.001) and age (*b* = − 0.995, S.E = 0.300, *p* = 0.002), with no main effect of sex (*b* = 0.024, S.E = 0.219 *p* = 0.914). There was no main effect of RF (*b* = − 0.215, S.E = 0.176, *p* = 0.227), and the association with 14-month AOSI did not reach significance (*b* = 0.045, S.E = 0.023 *p* = 0.056). However, the interaction between AOSI and RF was significant (*b* = − 0.107, S.E = 0.048, *p* = 0.031). To break this down, we ran regressions separately for high and low RF (based on a median split; see Fig. 1a, b), whilst controlling for risk group, age and sex. The AOSI was a significant predictor of SRS in those with low RF (*b* = 0.103, S.E = 0.034, *p* = 0.006) over and above the effect of risk group (*b* = 0.870, S.E = 0.256, *p* = 0.002) and age (*b* = − 0.783, S.E = 0.330, *p* = 0.025); the main effect of sex was not significant (*b* = 0.082, S.E = 0.268, *p* = 0.761). There was no association for those with high RF (*b* = 0.009, S.E = 0.033, *p* = 0.776). Risk group and age were both significant (*b* = 0.721, S.E = 0.313, *p* = 0.028; *b* = − 1.328, S.E = 0.525, *p* = 0.017, respectively) and sex was not (*b* = 0.144, S.E = 0.331, *p* = 0.667).

### Does RF moderate the association between infant activity level and later ADHD symptoms at 7 years?

**Fig. 2:**
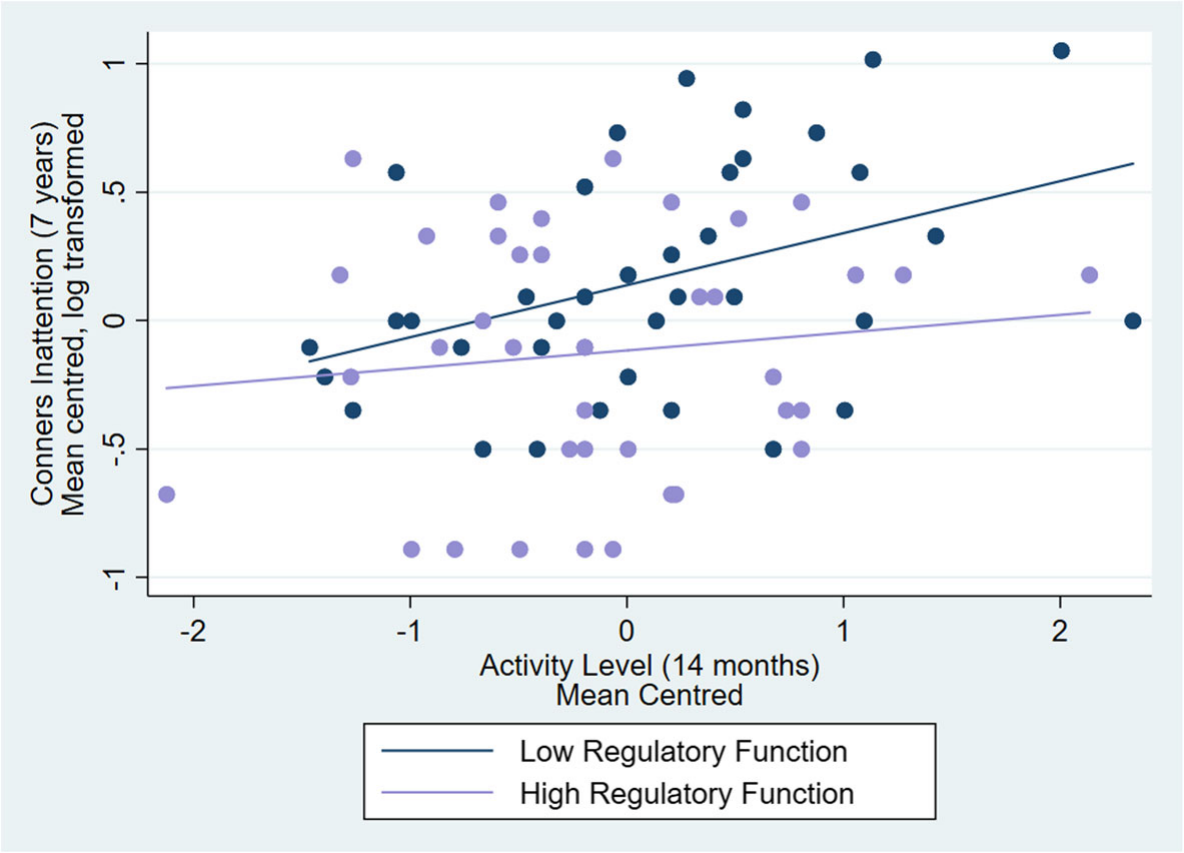
The effect of infant activity level predicting later ADHD inattention traits on the Conners for those with high versus low RF

For ADHD inattention, a multiple regression showed no significant associations between 7-year inattention score and either risk group (*b* = 0.193, S.E = 0.111, *p* = 0.087), age (*b* = − 0.199, S.E = 0.162, *p* = 0.225) or sex (*b* = 0.153, S.E = 0.121, *p* = 0.210). The main effect of RF (*b* = − 0.201, S.E = 0.108, *p* = 0.067) and 14-month activity (*b* = 0.126, S.E = 0.064, *p* = 0.054) did not reach significance. There was no significant interaction between activity and RF (*b* = 0.103, S.E = 0.077, *p* = 0.188). Although the interaction was not significant, as we had a specific a priori hypothesis about the association between activity score and ADHD traits depending on RF level, we ran the regressions separately for high and low RF. Activity was a significant predictor of inattention for those with low RF (*b* = 0.171, S.E = 0.081, *p* = 0.043, see Fig. 2) controlling for risk group (*b* = 0.138, S.E = 0.157, *p* = 0.387), age (*b* = − 0.182, S.E = 0.207, *p* = 0.386) and sex (*b* = 0.125, S.E = 0.159, *p* = 0.436). There was no association between activity and ADHD inattention for those with high RF (*b* = 0.069, S.E = 0.096, *p* = 0.477), and no significant effect of risk group (*b* = 0.209, S.E = 0.151, *p* = 0.175), age (*b* = − 0.296, S.E = 0.259, *p* = 0.262) and sex (*b* = 0.129, S.E = 0.173, *p* = 0.463).

For ADHD hyperactivity, a multiple regression showed no significant associations between 7-year hyperactivity score and either risk group (*b* = 0.251, S.E = 0.153, *p* = 0.106), age (*b* = − 0.377, S.E = 0.224, *p* = 0.097) or sex (*b* = 0.251, S.E = 0.167, *p* = 0.137). The main effect of RF was not significant (*b* = − 0.084, S.E = 0.149, *p* = 0.573), but there was a significant association with 14-month activity (*b* = 0.387, S.E = 0.089, *p* < 0.001). There was no significant interaction between activity and RF (*b* = − 0.024, S.E = 0.107, *p* = 0.826). When this was broken down by RF, for children with low RF, activity was a significant predictor of hyperactivity (*b* = 0.452, S.E = 0.126, *p* = 0.001) with no significant effect of risk group (*b* = 0.396, S.E = 0.243, *p* = 0.114), age (*b* = − 0.409, S.E = 0.321, *p* = 0.211) and sex (*b* = 0.238, S.E = 0.247, *p* = 0.343). Results were similar for high RF, with activity (*b* = 0.309, S.E = 0.119, *p* = 0.014) and not risk group (*b* = 0.094, S.E = 0.187, *p* = 0.617), age (*b* = − 0.449, S.E = 0.320, *p* = 0.170) or sex (*b* = 0.299, S.E = 0.214, *p* = 0.172) associated with later ADHD hyperactivity.

## Discussion

In line with the hypotheses presented earlier, we found evidence for a differential association between infant regulatory function (RF) and later disorder traits, with reduced RF significantly associated with later ASD and ADHD traits, but not with CU traits. Further, consistent with predictions from Johnson [5], we found evidence to support a protective role of strong RF within ASD, and to a lesser extent, ADHD traits: infant autism-like atypicality as measured by the AOSI was associated with later 7-year ASD traits only in those with low RF as infants. A similar effect was found for ADHD inattention traits; infant activity level was associated with later ADHD inattention only in those with low RF. However, for ADHD hyperactivity, activity level was significantly associated with later hyperactivity traits in both those with high and low EF.

The association between 14-month infant regulation and later traits of ASD at 7 years extends previous findings in the same sample in relation to 3-year ASD outcome [37]. Clifford et al. [37] found that children who went on to an ASD diagnosis at 3 years had lower levels of regulation (termed effortful control in their paper as it was measured at both 14 months and later in development at 24 months, when inhibitory control is included in the subscale). Whilst there is a good degree of stability in clinical diagnosis from toddlerhood to mid-childhood in infants at risk for ASD [52, 62], there is nevertheless still change with some children only meeting diagnostic criteria at a later age and others meeting criteria as toddlers but not in mid-childhood. It is useful to know whether the same infant markers remain predictive later in development (e.g., [63]), and future studies with larger samples should test whether infant predictors such as regulation abilities can discriminate stable cases from those who shift diagnostic categories or show a great degree of change in symptom severity.

We also found a significant association between regulatory function and traits of ADHD inattention, suggesting that this effect is not ASD specific. For ADHD hyperactivity, however, the correlation with early RF did not reach significance (although it is worth noting that the beta was the same as for inattention, so this will need to be replicated in larger samples). These findings are consistent with previous studies showing that the hyperactive-impulsive subtype of ADHD is not strongly associated with EF difficulties (e.g., [64, 65]). Indeed, Willcutt et al. [4] in a review of the literature suggested that EF weaknesses may be associated predominately with inattention symptoms.

In contrast to ASD and ADHD inattention, no association was found between infant RF and later CU traits. The difference in the association between RF and later CU traits compared to both ASD and ADHD inattention traits approached significance. This is consistent with previous findings that, unlike in other developmental disorders [2], EFs do not appear to be impaired in psychopathic or CU traits [19, 66, 67]. This may be because there are differential associations between different subtypes of CU, with impaired EF in those children who show a profile of CU traits with high anxiety, more akin to secondary psychopathy, but enhanced EF in those with primary psychopathy traits [68]. In future studies, characterising the specific associations between EF and different ‘constructs’ [69] such as attention and social communication, rather than just the clinical disorders or symptoms, will be important. This will allow us to make specific predictions about how the presence of a co-occurring disorder, with a different profile of strengths and weaknesses, might change performance for a given construct or task (e.g., [19, 70]).

Our second aim, following the hypothesis put forward by Johnson [5], was to test whether high executive functions—or in this case regulatory functions, as a precursor of executive function—could act as a protective factor. We found evidence that early behavioural signs measured by the AOSI were only associated with later ASD symptoms in those with low RF. Johnson [5] suggests that strong EF may play a protective role by modulating neural activity according to the capacity of posterior brain regions, i.e., selecting the best combination of computational regions for a given learning problem. How might this translate to the behavioural level? One possibility is that it enables more flexible use of compensatory strategies. In ASD, strong RF could help mitigate the impact of early markers (in this case early autism-like behavioural atypicalities) via multiple possible mechanisms. For example, learning and remembering to make eye contact and explicit emotion understanding/theory of mind through logical reasoning [71]. Related to this, strong RF might be better characterised as masking rather than truly reducing autistic traits—it is possible that the lower SRS scores indicate an increased ability to regulate oneself around others. This type of compensation may be ‘shallow’ which Livingston and Happé [71] describe as being more effortful and prone to break down under stress.

For ADHD traits, infant activity level was associated with later ADHD inattention in those with low but not high RF. Children showing high infant activity levels but also strong EFs may have a greater ability to choose when and what to pay attention to, resulting in lower inattentive traits later in development. For ADHD hyperactivity, on the other hand, there was no evidence of moderation by RF, with activity level predicting later hyperactivity irrespective of RF. This may be because whilst children with high RF are able to control their behaviour at times, for example when concentrating on a task, they continue to manifest hyperactivity at other times, which results in parents forming a global opinion of activity levels being high. To test this hypothesis, it would be important for future studies to use objective measures of activity, such as accelerometer data.

Whilst the current study set out to test a moderating role of EF, based on the hypothesis by Johnson [5], it is important to acknowledge that at both a statistical and conceptual level, it is also possible that rather than enhanced RF acting as a protective factor, low RF is acting as an additional risk factor, compounding the effects of other early markers of atypicality [72]. Future work should test the cross-lagged effect of infant markers and EF across multiple time points, to clarify the temporal order of effects. However, even with a clear temporal ordering of putative risk and protective factors, it is worth noting that any cognitive protective (or risk) factor may represent a precursor to later symptoms which is, therefore, not independent of outcome. Whilst this possibility remains important to consider, we argue that evidence in favour of RF as a protective/risk factor also includes the fact the RF shows cross-domain (i.e., for social communication and restricted and repetitive behaviours, see Additional file 1) and cross-disorder (i.e., ADHD) influences.

The current sample includes both high- and low-risk infants. Whilst the main effect of risk group is included in the analysis, the relatively modest sample size precludes testing more complex models with interactions between group and RF, as well as 3-way group × RF × infant marker interactions. It seems likely that the protective role of RF may work differently in those with and without familial ASD risk, and future studies should test this explicitly. Future more highly powered studies should also investigate the potential moderating role of sex. Bedford et al. (2016) [73] demonstrated that whilst there were no significant sex differences in several infant markers for ASD (including the AOSI score used in the current paper), the association between infant markers and disorder traits was moderated by sex; the association was only significant for boys. Whilst the current study controlled for a main effect of sex, future, more highly powered studies should investigate whether apparent protective effects of biological sex could be explained by increased regulatory function in girls [74].

Another limitation is the use of parent report measures for disorder traits, which likely share measurement error and could have been subject to rater bias. However, such ‘halo effects’, where parents form a global impression of a child and consequently rate them in a similar way across multiple domains, would be more likely to result in similar associations across all disorder traits, which we did not find. Future studies, with larger sample sizes, should use structural equation modelling approaches, such as bifactor models, to attempt to separate out the shared and distinct variance across disorder traits. Whilst an experimenter-led observational measure was used for early infant autism behaviours (AOSI), activity level was also parent rated, from the IBQ-R. It was chosen because it is the only available infant marker (i.e., in 14-month-olds) that has been shown to specifically predict later ADHD traits, but as noted above, future studies should replicate this with objective measures such as accelerometer data.

These results have potentially significant implications when thinking about targeting EF in interventions. Executive function difficulties are related to lower adaptive functioning in both ASD [12, 75] and ADHD [76]. Thus, interventions to support the optimal development of EF may have significant benefits across ASD and ADHD, although of course, we demonstrate no causality in the current study. Promisingly, there is some evidence for the potentially modifiable nature of EF following intervention programs (e.g., [77]), particularly early in development when the brain is most plastic [5, 78]. Given that EF interventions may act to improve a variety of symptoms, they have the potential to offer more widespread cross-domain benefits than disorder-specific interventions.

## Conclusions

In conclusion, our findings showed an association between infants’ regulation ability and both ASD and ADHD inattention traits in mid-childhood. We also found suggestive evidence in support of the hypothesis that strong regulation abilities (RF; a precursor to executive function) may have a protective effect across developmental disorders [5]. Early markers for ASD (autism-like behaviours) and ADHD inattention (activity level) were only associated with later disorder traits in children with low RF. This suggests that having strong RFs could potentially allow children to compensate for additional neural or behavioural atypicalities. However, this association with later developmental disorder traits was not universal, with no association for later ADHD hyperactivity or CU traits. Future research is needed to establish which additional factors influence when strong EFs are protective and when they are not.

## Additional file

Additional file 1: Supplementary Materials. (DOCX 24 kb)

## Data Availability

The datasets used and/or analysed during the current study are available from the BASIS study on reasonable request.

## Abbreviations

ADHD: Attention deficit/hyperactivity disorder
ADI-R: Autism Diagnostic Interview—Revised
ADOS-2: Autism Diagnostic Observational Schedule*—*Second Edition
ADOS-G: Autism Diagnostic Observational Schedule*—*Generic
AOSI: Autism Observation Scale for Infants
ASD: Autism spectrum disorder
BAP: Broader autism phenotype
BASIS: British Autism Study of Infant Siblings
CU: Callous unemotional
DAWBA: Development and Well-Being Assessment
DSM-5: Diagnostic and Statistical Manual 5th edition
EF: Executive function
IBQ-R: Infant Behavior Questionnaire—Revised
ICU: Inventory of Callous Unemotional Traits
RCO: Regulatory capacity/orienting
RF: Regulatory function
SCQ: Social Communication Questionnaire
SRS-2: Social Responsiveness Scale—Second Edition
VABS-II: Vineland Adaptive Behavior Scales—Second Edition

## Footnotes

1 5 DAWBA and 5 SCQ missing.
