# Supplementary material for: Infant regulatory function acts as a protective factor for later traits of autism spectrum disorder and attention deficit/hyperactivity disorder but not callous unemotional traits
Source: J Neurodev Disord. 2019 Jul 18;11:14. doi: 10.1186/s11689-019-9274-0 (PMC6660706; doi:10.1186/s11689-019-9274-0)
Supplement: Supplementary file 1 — Supplementary Materials. (DOCX 24 kb) [file 11689_2019_9274_MOESM1_ESM.docx]

**Table S1** Group characteristics and group means for symptoms in mid-childhood (age 7 years). Means (SD) by HR and LR group.

|  | **HR group** | **LR group** | **Group differences** |
| --- | --- | --- | --- |
| ***7-year measures*** |  |  |  |
| Sex, N girls: N boys | 28:16 | 22:15 | n/s |
| Age, months  *N (girls)* | 90.81 (6.33)  *43 (27)* | 89.34 (4.81)  *35 (21)* | n/s |
| Conners Inattention  *N (girls)* | 57.07 (13.95)  *42 (26)* | 51.22 (9.40)  *37 (22)* | *t* (72.30) = -2.21, *p* = .03 |
| Conners Hyp/Imp  *N (girls)* | 59.26 (16.59)  *42 (26)* | 52.16 (11.58)  *37 (22)* | *t* (73.35) = -2.23, *p* = .03 |
| ICU Total Score  *N (girls)* | 21.83 (11.27)  *40 (26)* | 18.09 (5.96)  *32 (19)* | n/s |
| SRS-2 T-score  *N (girls)* | 59.27 (19.63)  *37 (23)* | 45.49 (5.82)  *35 (21)* | *t* (42.62) = -4.09, *p* < .001 |
| WASI-II FSIQ  *N (girls)* | 109.34 (16.29)  *41 (27)* | 117.06 (11.61)  *35 (21)* | *t* (74) = 2.34, *p* = .02 |

*N (girls) =* the number of participants in each group that completed each measure and the number of children completing the measure that were girls. *Conners Inattention and Hyp/Imp* = Conners 3 T-scores for DSM-IV Inattentive and Hyperactive/Impulsive domains. *SRS-2* = Social Responsiveness Scale – 2. *WASI-II FSIQ* = Wechsler Abbreviated Scale of Intelligence – 2^nd^ Edition full scale IQ. Note that corrected df and *p*-values are reported wherever the assumption of equal variances was violated in t-tests.

*Correlations between Regulatory Function (RF) and ASD and CU subscales.*

Correlations use the same variables as those analysed in the models below.

There was a significant negative correlation between centred RF and centred, skew-transformed Social Communication and Interaction (SC) subscale of the SRS: r = -0.336, p = 0.005, with a similar trend which did not reach significance for Restricted Interests and Repetitive Behavior (RRB): r = -0.220, p = 0.069.

Partial correlations between the centred, skew-transformed ICU subscales and centred RF controlling for risk group were all non-significant, callous: r = 0.161, p = 0.170, unemotional: r = -0.091, p = 0.458 and uncaring: r = -0.058, p = 0.635.

*Supplementary statistical analysis*

We ran several post-hoc analysis. First, to determine whether results were similar for different ASD trait domains, we ran separate regression models for SRS Restricted Interests and Repetitive Behavior (RRB) and Social Communication and Interaction (SC) subscales. As in the main paper, an lnskew0 transformation was applied to outcome variables, which showed a non-significant deviation from normal for SRS SC (p = 0.44) but not for SRS RRBs (p < 0.001). We therefore ran the RRB regression with bootstrapping and 1000 repetitions. Second, we used ASD traits from the SCQ to check whether findings were specific to the SRS. The SCQ distribution was not normal even following lnskew0 transformation, so again bootstrapped regression results (1000 repetitions) are presented. Finally, we re-ran the analyses in the main paper without risk group as a covariate.

1. *SRS Restricted Interests and Repetitive Behavior (RRB) and Social Communication and Interaction (SC) subscales*

**Table S2a: Regression results for SRS Restricted Interests and Repetitive Behavior (RRB)**

|  | SRS RRBs full model  b (bootstrapped SE), p | Low RF  b (bootstrapped SE), p | High RF  b (bootstrapped SE), p |
| --- | --- | --- | --- |
| AOSI | 0.09 (0.07), p = 0.20 | 0.20 (0.11), p = 0.06 | 0.01 (0.10), p = 0.95 |
| RF | -0.32 (0.56), p = 0.57 | - | - |
| AOSI*RF interaction | -0.28 (0.16), p = 0.08 | - | - |
| Group | **1.45 (0.64), p = 0.02** | **2.81 (0.83), p = 0.001** | 0.63 (0.86), p = 0.47 |
| Age | **-2.60 (0.94), p = 0.006** | -2.21 (1.33), p = 0.10 | **-3.46 (1.31), p = 0.008** |
| Sex | -0.10 (0.73), p = 0.90 | -0.01 (1.10), p = 0.99 | 0.19 (0.93), p = 0.84 |

**Table S2b: Regression results for SRS Social Communication and Interaction (SC)**

|  | SRS SC full model  b (bootstrapped SE), p | Low RF  b (bootstrapped SE), p | High RF  b (bootstrapped SE), p |
| --- | --- | --- | --- |
| AOSI | 0.03 (0.02), p = 0.23 | **0.08 (0.03), p = 0.02** | -0.01 (0.03), p = 0.83 |
| RF | -0.24 (0.17), p = 0.15 | - | - |
| AOSI*RF interaction | **-0.10 (0.05), p = 0.03** | - | - |
| Group | **0.72 (0.20), p = 0.001** | **0.84 (0.24), p = 0.001** | **0.63 (0.30), p = 0.045** |
| Age | **-0.81 (0.28), p = 0.006** | **-0.67 (0.31), p = 0.04** | **-1.12 (0.51), p = 0.03** |
| Sex | -0.03 (0.21), p = 0.90 | -0.09 (0.25), p = 0.72 | 0.17 (0.32), p = 0.59 |

Tables S2a&b indicate the pattern of results was similar for RRBs and SC subscales.

1. *ASD traits measured via Social Communication Questionnaire (SCQ)*

**Table S3: Regression results for Social Communication Questionnaire (SCQ)**

|  | SCQ full model  b (bootstrapped SE), p | Low RF  b (bootstrapped SE), p | High RF  b (bootstrapped SE), p |
| --- | --- | --- | --- |
| AOSI | 0.05 (0.03), p = 0.12 | **0.14 (0.05), p = 0.009** | 0.01 (0.05), p = 0.89 |
| RF | -0.23 (0.35), p = 0.51 | - | - |
| AOSI*RF interaction | **-0.18 (0.09), p = 0.04** | - | - |
| Group | 0.63 (0.35), p = 0.07 | **1.54 (0.50), p = 0.002** | -0.26 (0.47), p = 0.58 |
| Age | -0.93 (0.55), p = 0.09 | **-1.23 (0.61), p = 0.04** | -0.91 (0.78), p = 0.24 |
| Sex | -0.48 (0.36), p = 0.19 | -0.57 (0.49), p = 0.24 | -0.08 (0.46), p = 0.87 |

Table S3 shows results for the SCQ are highly similar to that seen for the SRS.

1. *Regression models without risk group as a covariate*

**Table S4a: Regression results for SRS**

|  | SRS full model  b (bootstrapped SE), p | Low RF  b (bootstrapped SE), p | High RF  b (bootstrapped SE), p |
| --- | --- | --- | --- |
| AOSI | **0.06 (0.03), p = 0.02** | **0.11 (0.04), p = 0.008** | 0.02 (0.03), p = 0.48 |
| RF | **-0.48 (0.18), p = 0.009** | - | - |
| AOSI*RF interaction | -0.10 (0.05), p = 0.08 | - | - |
| Age | **-0.87 (0.33), p = 0.01** | -0.64 (0.39), p = 0.11 | **-1.35 (0.56), p = 0.02** |
| Sex | 0.11 (0.24), p = 0.64 | -0.09 (0.32), p = 0.78 | 0.23 (0.35), p = 0.52 |

**Table S4b: Regression results for ADHD Inattention**

|  | Inattention full model  b (bootstrapped SE), p | Low RF  b (bootstrapped SE), p | High RF  b (bootstrapped SE), p |
| --- | --- | --- | --- |
| Activity | **0.13 (0.07), p = 0.05** | **0.18 (0.08), p = 0.03** | 0.07 (0.10), p = 0.46 |
| RF | **-0.26 (0.10), p = 0.02** | - | - |
| Activity*RF interaction | 0.11 (0.08), p = 0.17 | - | - |
| Age | -0.15 (0.16), p = 0.35 | -0.14 (0.20), p = 0.49 | -0.29 (0.26), p = 0.28 |
| Sex | 0.18 (0.12), p = 0.14 | 0.14 (0.16), p = 0.40 | 0.16 (0.17), p = 0.36 |

**Table S4c: Regression results for ADHD Hyperactivity/Impulsivity**

|  | Hyp/Imp full model  b (bootstrapped SE), p | Low RF  b (bootstrapped SE), p | High RF  b (bootstrapped SE), p |
| --- | --- | --- | --- |
| Activity | **0.39 (0.09), p < 0.001** | **0.48 (0.13), p = 0.001** | **0.31 (0.12), p = 0.01** |
| RF | -0.16 (0.14), p = 0.27 | - | - |
| Activity*RF interaction | -0.02 (0.11), p = 0.89 | - | - |
| Age | -0.32 (0.22), p = 0.16 | -0.29 (0.32), p = 0.38 | -0.45 (0.32), p = 0.17 |
| Sex | 0.29 (0.17), p = 0.09 | 0.27 (0.25), p = 0.30 | 0.31 (0.21), p = 0.14 |
